# American Society of Clinical Oncology Multidisciplinary Cancer Management Course: Connecting Lives, Cancer Care, Education, and Compassion in Zimbabwe—A Pilot for Efforts of Sustainable Benefit?

**DOI:** 10.1200/JGO.2016.003673

**Published:** 2016-09-14

**Authors:** Sandra Ndarukwa, Anna Mary Nyakabau, Anees B. Chagpar, David Raben, Ntokozo Ndlovu, Webster Kadzatsa, Vanessa J. Eaton, Paida Mafunda, Evangelia Razis

**Affiliations:** **Sandra Ndarukwa** and **Anna Mary Nyakabau**, Parirenyatwa Group of Hospitals; **Ntokozo Ndlovu** and **Webster Kadzatsa**, University of Zimbabwe College of Health Science, Harare; **Paida Mafunda**, Junior Chamber International Zimbabwe, Harare, Zimbabwe; **Anees B. Chagpar**, Yale University, New Haven, CT; **David Raben**, University of Colorado School of Medicine, Denver, CO; **Vanessa J. Eaton**, American Society of Clinical Oncology, Alexandria, VA; and **Evangelia Razis**, Hygeia Hospital, Athens, Greece.

## Abstract

The burden of cancer in low- to middle-income countries is growing and is expected to rise dramatically while resources to manage this disease remain inadequate. All authorities for the management of cancer recommend multidisciplinary care. Educational efforts by international organizations to assist local professionals in caring for their patients tend to have a lasting impact because they empower local professionals and enhance their skills. A multidisciplinary cancer management course was designed by American Society of Clinical Oncology staff and local experts to provide a roadmap for cross-specialty interaction and coordination of care in Zimbabwe. The outcome of the course was measured through feedback obtained from participants and impact on local workforce. The cancer management course was relevant to daily practice and fostered long-lasting partnerships and collaborations. Furthermore, it resulted in a more motivated local workforce and strengthened existing multidisciplinary practices. Cancer care is in a critical state in low- to middle-income countries. Educational efforts and collaborative partnerships may provide a cost-effective strategy with sustainable benefits. A multidisciplinary approach to optimize therapy is desirable. Evaluation of the course impact after a period of 6 months to 1 year is needed to determine the sustainability and impact of such efforts.

## INTRODUCTION

The burden of cancer in low- and middle-income countries (LMICs) is increasingly a global concern. The incidence of cancer is expected to rise dramatically in these regions, while the resources to manage this disease remain inadequate.^1,2^ Several organizations are engaging in volunteering efforts whereby physicians travel from high-income to low-income countries to assist local professionals in caring for their patients. These efforts, although well intentioned, are generally short lived, and their impact is often unsustainable.^3-7^ Educational efforts, facilitating transfer and sharing of knowledge, may have a more lasting impact, although there may be factors that influence the success of such measures.^8,9^ Multidisciplinary care has been widely adopted as the standard of care in cancer management, with studies showing significant changes in diagnoses and treatment plans.^10-12^ However, data on the impact of multidisciplinary care in developing countries are limited.

Zimbabwe is a country located in the sub-Saharan region of Africa. It has a population of 13,061,239 (according to the 2012 national census), with a ratio of approximately 93 males to 100 females.^13^ The average life expectancy is approximately 58 years.^14^ Its gross domestic product as of 2014 was US$14.2 billion.^15^ The country has been greatly affected by the HIV pandemic, with 15% of adults infected by the virus.^16^ This has also affected the epidemiology of cancers in Zimbabwe, with HIV-related cancers comprising 60% of new cases of cancer per year, as noted by the Zimbabwe National Cancer Registry (NCRZ).^17^ The major diseases in Zimbabwe are HIV and AIDS, tuberculosis, and malaria.^18^ Zimbabwe is now facing the threat of noncommunicable diseases like cardiovascular disease, diabetes, and cancer, particularly cervical cancer.^19^ The density of physicians per population of 1,000 was 0.07 as of 2009.^20^

The health care system in Zimbabwe can be described as recovering after a decline in the first decade of this millennium.^21^ This was the result of a deterioration in infrastructure, poor investments, poor remuneration for health workers, and a shortage of essential supplies and commodities, especially in late 2008 and early 2009, that led to the near collapse of the health sector. In 2009, the government developed the National Health Strategy, which sought to reverse the decline in the performance of the delivery system.^22^ This included increasing the level of health financing, improving access to basic medicines and equipment, taking steps to attract and retain health workers, and laying foundations for investment policy to rehabilitate the health sector.

The public health system is the largest provider of health services, complemented by mission hospitals and nongovernmental organizations. The government has introduced a user fee, whereby patients pay for services at health institutions. These fees are now providing income for many health care facilities, enabling them to provide at least minimum-level services.

Cancer in Zimbabwe is characterized by poor survival rates. This is mainly a result of advanced stages of cancer at presentation, with 80% of patients presenting with stage III or IV disease.^23^ A study by Gondos et al^24^ in 2008 on cancer survival in Zimbabwe showed that cancer survival among black Zimbabweans was low, with no survival estimate exceeding 55% at 5 years. A study investigating survival from the most common cancers diagnosed in the period from 2004 to 2008 is currently under way.^25^

The NCRZ is one of the few functional cancer registries in Africa, having been established in 1985 as a result of a collaborative research agreement between the Ministry of Health and the WHO International Agency for Research on Cancer. It is a population-based registry based in a tertiary hospital covering all 10 provinces of Zimbabwe. It has contributed data to four successive editions of Cancer Incidence in Five Continents, with numerous publications in peer-reviewed medical journals. Data from the NCRZ are used in the GLOBOCAN series of publications for estimates on incidence and mortality.

## CANCER BURDEN IN ZIMBABWE

**Fig 1:**
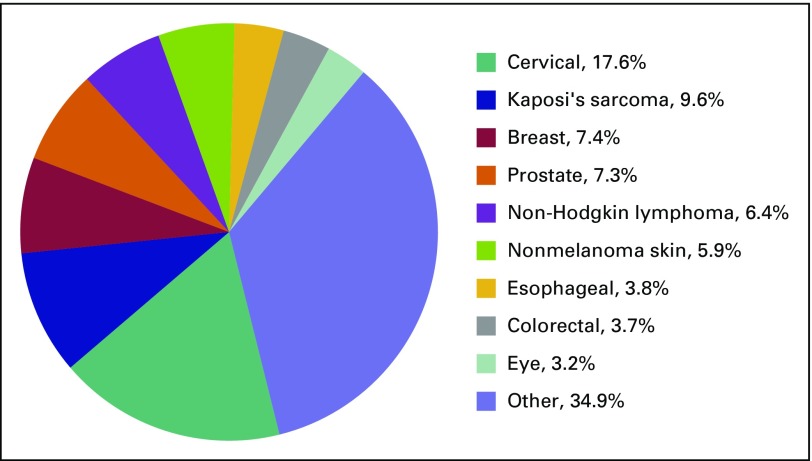
Cancer incidence in Zimbabwe according to Zimbabwe Cancer Registry data from 2005 to 2013.

**Fig 2:**
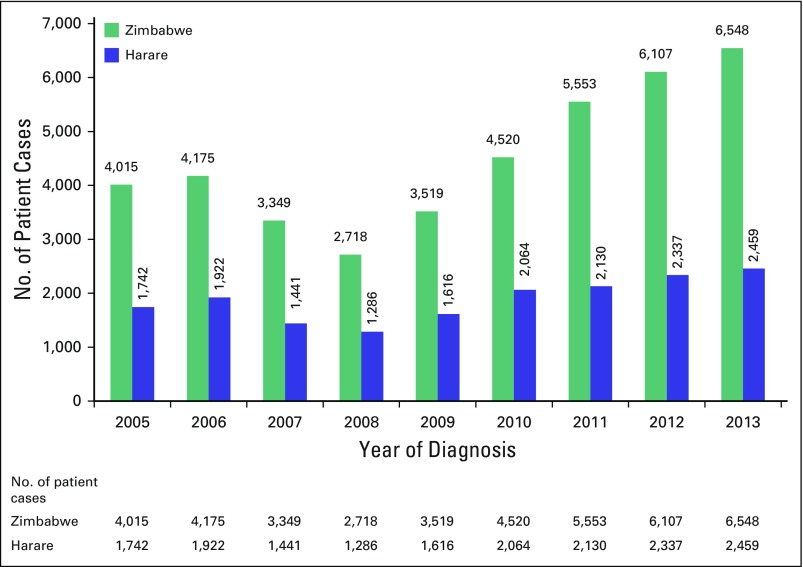
Cancer incidence in Zimbabwe according to Zimbabwe Cancer Registry 2013 Annual Report.^17^

From 2008 to 2013, the number of cancer cases in Zimbabwe increased from 2,718 to 6,548 (Fig 1).^17^ It remains unclear as to whether this increase resulted from higher rates of HIV burden and the more widespread adoption of a Western lifestyle, with the associated cancer risk factors related to increasing obesity and smoking, or whether it was the result of more cases being diagnosed and recorded, with raised awareness and improved access to health facilities.^26^ Infection-related cancers (resulting from HIV and human papillomavirus) continue to dominate (Fig 2). However, the WHO has predicted a sharp increase in new cases globally, mainly because of aging populations, current trends in smoking prevalence, and a growing adoption of unhealthy lifestyles.^2^

Of the 6,548 cases reported by the NCRZ in its 2013 annual report, approximately 42.4% occurred in men.^6^ The most frequently occurring cancers for all races were: cervical cancer (18%), Kaposi's sarcoma (10%), breast cancer (7%), prostate cancer (7%), non-Hodgkin lymphoma (6%), skin cancer (6%), GI tract cancer (8%), and eye cancer (3%). Of particular concern is that a majority of patients (81%) present with stage III or IV disease.^6^ Figure 2 shows cancer incidence in Zimbabwe according to the NCRZ 2013 annual report.

## CHALLENGES IN CANCER CARE DELIVERY

There are two state-owned cancer treatment centers in Zimbabwe: one at Parirenyatwa Group of Hospitals in the capital city of Harare and the other at Mpilo Central Hospital, Bulawayo, situated in the southwestern part of the country. At these centers, there are chemotherapy, radiotherapy, and nuclear medicine facilities. There are eight qualified clinical oncologists, with six working at the main center in Harare and two in Bulawayo. There are 10 registrars at various levels of training in the radiotherapy and oncology masters degree program at the University of Zimbabwe. The Zimbabwean model of radiotherapy and oncology training for physicians is based on the clinical oncology model, where training covers both radiation oncology and medical oncology for a minimum 4-year period.

There are approximately 1,500 new patient cases seen per year at the main radiotherapy center in Harare, resulting in a ratio of oncologists to patients of one to 250. The Mpilo radiotherapy center sees approximately 500 new patient cases per year. The GLOBOCAN report on human resources for treating new cancer cases in Zimbabwe estimated that Harare needed eight radiation or clinical oncologists, and Bulawayo needed four oncologists.^27^ These estimates are not far from the numbers that already exist in Zimbabwe, and the training program will ensure these numbers continue to increase.

Although these facilities exist, cancer care delivery in Zimbabwe remains a challenge. Zimbabwe has always given priority to cancer control in its national health care plan. This is guided by the 2009 to 2013 National Health Strategy, which has been expanded to cover 2016. The main focus of this strategy is quality and equity in health to improve the quality of life for Zimbabweans.^22^ However, because of the burden of communicable diseases, especially HIV and AIDS and malaria, priority has been given to combating these diseases. The response of national systems to noncommunicable diseases is underdeveloped.^19^ Funding constraints have left noncommunicable diseases like cancer underfunded.

The referral system for patients is not fully functional, with a lack of skilled professionals at district and provincial levels and specialized services available only at tertiary institutions. This makes it difficult for the majority of patients to access cancer services. The cost of these services has also been prohibitive. All services are paid for with either cash or medical insurance coverage. A majority of patients who need care are unemployed, with most coming from the rural areas. The prohibitive cost of traveling to a center of care and receiving treatment results in most patients failing to access treatment.

As is the case in other low-income countries, Zimbabwe has been negatively affected by the brain drain of skilled personnel, with the education and health sectors most affected. A study by Clemens and Petterson^28^ found that 51% of Zimbabwean physicians and 24% of nurses were estimated to be working elsewhere in the world. The government has responded by establishing policies encouraging retention of these professionals. This includes providing housing and transportation allowances for staff. The salaries and on-call allowances of critical staff have been increased. The government has also introduced fellowship and scholarship programs and advanced training programs to improve the skills of health professionals.^29^ A staff retention policy for government-trained physicians and nurses has been introduced, where staff are retained in government service for the number of years trained.

Another challenge is inadequate resources to maintain and improve the cancer treatment facilities available in the country. The two centers in Harare and Bulawayo have state-of-the-art equipment, including five linear accelerators, two simulator machines, and three brachytherapy machines; however, these need regular servicing and repair. More machines need to be acquired to meet the International Atomic Energy Agency (IAEA) recommendation of one machine per 1 million people in a developing country in Africa.^30^ Reactivation of the nuclear medicine departments, which have been nonfunctional since 2003, at both centers is also required.

There is no comprehensive national cancer control program in Zimbabwe.^31^ Efforts are under way to address this through the strengthening of local training programs and the recent adoption of the National Cancer Control Strategy, which is aligned with the National Health Strategy.^23^ The main goal of the initiative is to coordinate cancer management from a central point to develop policies that ensure preventive treatment and palliative care services are delivered, in an effort improve the low cancer survival rates. The strategy was launched in July 2013, and a task force has been established to evaluate and monitor the performance of the program and advise the Ministry of Health.^23^

## MULTIDISCIPLINARY CARE

Managing patients with cancer is becoming complex and requires a multidisciplinary approach.^32^ Multidisciplinary management of patients offers the potential benefit of having physicians of different specialties participating in treatment planning. This may help in the establishment and use of common clinical guidelines, thus streamlining care and establishing a minimum level of quality.^33,34^ In turn, the streamlining of care could result in cost and time savings, decreased wastage of resources, and improvement in the value of patient care.^34^ There are limited studies evaluating the influence of multidisciplinary care on decision making and patient outcomes. Single-center studies have reported significant changes in diagnosis and treatment plans.^10,12^ A recent multi-institutional survey of US Veterans Affairs found limited association between tumor boards, care, and outcomes.^35^

Multidisciplinary management includes multidisciplinary clinics, multidisciplinary team meetings or tumor boards, and mini tumor boards.^34^ These are held regularly, often once per week, and attended by key specialists involved in cancer management, who discuss the diagnosis and management of the patient with cancer.^34^ Multidisciplinary team meetings usually occur once per week and are attended by a core team of oncologists, surgeons, radiologists, pathologists, and other specialists, depending on the type and specialty of the tumor board.^36,37^ Mini tumor boards tend to be smaller groups of specialists, occurring where there are not enough specialists, as in resource-limited countries.^34,37^

Global tumor boards, which are live online telemedicine discussions of patient-based clinical scenarios between oncologists in LMICs and cancer experts in developed nations, are another way multidisciplinary teams can engage. These have assisted in situations where physicians lack the time and resources to keep up to date on current international practice guidelines or work in institutions that lack key specialists.^38^

Data from LMICs, although limited, have shown the effectiveness of multidisciplinary cancer care. Tumor board meetings may be important in limited-resource settings, where specialists may be less available, resulting in suboptimal care delivery.^34,36^ Evidence has shown that limitations in diagnosis and management can be overcome in suboptimal settings in rural or low-resource areas through presentation of clinical cases at multidisciplinary meetings or tumor boards.^39^ A study examining the use of multidisciplinary management tumor boards in Arab countries showed the importance and usefulness of tumor board meetings; it also indicated that in the absence of complete multidisciplinary teams, mini tumor boards are helpful.^34^ This study noted that in areas with limited resources, where not all experts and subspecialties are available, mini tumor boards enabling radiologists, pathologists, surgeons, and medical oncologists to meet can be appropriate and better than no boards at all. A report by the Breast Health Global Initiative on optimization of breast cancer management in low- and middle-resource countries identified challenges faced in these countries, including the lack of multidisciplinary care practice.^40^ A similar article by Saghir et al^41^ noted the relevance of this practice in low-resource countries and reported that most of these countires lacked the necessary health care system infrastructure to support multidisciplinary care.^41^ El Saghir et al^37^ reported that physicians in LMICs planning to establish tumor boards could benefit from the experiences of their colleagues already practicing multidisciplinary tumor boards, ensuring they organize more efficient tumor boards.^37^

In Zimbabwe, the multidisciplinary cancer care approach is slowly being adopted in an effort to better coordinate care and communication for patients across different disease sites. Because of the challenges of patient burden and inadequate personnel, multidisciplinary meetings and clinics are not held as regularly as they are at centers abroad. Tumor board meetings are held either once per week or once per month. Established tumor board meetings include weekly gynecology tumor boards, with general surgery and head and neck tumor board meetings held monthly. The meetings tend to take the form of mini tumor boards, because specialists representing all areas of patient care are not always available. The multidisciplinary meetings tend to be for selected patients who have already received some form of management. A breast multidisciplinary clinic is held once per week in the surgical wards. This is done for all new patients. Surgeons and oncologists meet to discuss individual patient cases.

Telemedicine discussions are held regularly, with the Parirenyatwa Group of Hospitals being a part of the Pan African e-Network project, which connects members with Indian experts in various areas of medicine, including oncology, through continuing medical education programs.^42^ The Department of Radiotherapy and Oncology at Parirenyatwa Hospital also holds monthly Afronet teleconference tumor board meetings, where oncologists from various African countries present challenging patient case scenarios for discussion with a panel of experts from Vienna^43^ The department is in the process of connecting with the Global Breast Tumor Board, organized by the Global Cancer Institute, which connects physicians around the world to discuss complex patient cases.^38^

As more local professionals are trained in different oncology specialties, meetings will develop into full tumor board meetings, and thus, patients will experience the full benefits of multidisciplinary care. Collaborative partnerships have been formed between the Ministry of Health and Child Care and United Nations organizations, such as the IAEA and the United Nations Development Program, which provide technical and financial support for cancer care in Zimbabwe. The IAEA in particular has been heavily involved in the local training of personnel and in the facilitation of fellowship awards to oncology professionals for training abroad. The IAEA has also provided technical and financial support toward the acquisition, installation, and maintenance of radiotherapy equipment.^44^

## MULTIDISCIPLINARY CANCER MANAGEMENT COURSE

The first international multidisciplinary cancer management course in Zimbabwe, designed to provide a roadmap for cross-specialty interactions and coordination of care, took place in Harare from August 31, 2015, to September 4, 2015. It was a collaborative effort between the American Society of Clinical Oncology (ASCO) and the Association of Radiation Oncologists and Radiologists of Zimbabwe through the University of Zimbabwe College of Health Sciences. ASCO facilitated and supported experts in breast, head and neck, and colorectal cancers to come and deliver educational seminars and also provide expert clinical advice.

Premeeting lectures delivered included a lecture on glioblastomas attended by oncologists and local neurosurgeons. Didactic lectures on up-to-date management of head and neck and breast cancers were given to surgical and oncology professionals. The faculty also participated in a head and neck tumor board meeting and had valuable hands-on discussions with the radiotherapy team. The visiting breast surgeon was also able to join local surgeons in the operating theater, where she assisted with surgical techniques in managing breast masses. Grand rounds were also performed in oncology wards.

The cancer management course focused on breast, colorectal, and head and neck cancers, in an effort to review and discuss current standards of care and the advantages of a multidisciplinary approach in these tumors. More than 120 health care professionals, mostly specialists from an array of disciplines, attended the course from Zimbabwe and surrounding countries. The multidisciplinary nature of the faculty ensured that all aspects of breast, colorectal, brain, and head and neck cancer management were discussed.

A so-called train the trainer course was facilitated after the workshop. The purpose of the course was to help establish multidisciplinary care teams and guidelines in local hospital facilities. Participants were from different regions of the country and also from diverse backgrounds; they included specialist physicians, pharmacists, and members of the Cancer Association of Zimbabwe and Island Hospice of Zimbabwe. All participants committed to instituting multidisciplinary teams in their hospitals. The lecture content was made available to all participants.

### Course Evaluation

A postcourse evaluation was conducted by ASCO Multidisciplinary Cancer Management Course organizers on site as well as through an online survey shortly after the conclusion of the course; results are summarized in Table 1. Only 24% (29 of 120) of the attendees completed the evaluation forms on day one; 10% (13 of 120) completed them on day two of the course. The feedback was quite consistent. The overall impression was positive, with some participants suggesting that the course should be repeated on a regular basis. A majority of the participants (79% of respondents) indicated a commitment to making practice changes after the course. These changes included adopting a multidisciplinary approach to care, improving interspecialty communication, and increasing the number of referrals to other specialties. The participants listed as potential barriers to change the lack of resources, lack of support from other colleagues, lack of staff, and lack of time. On average, 80% of respondents felt they learned new skills in diagnosis and treatment and were more confident in their ability to manage the cancers covered in the course. Some participants would have preferred a less technical approach, for example, lectures at a level understandable to attendees from all specialties.

**Table 1:**
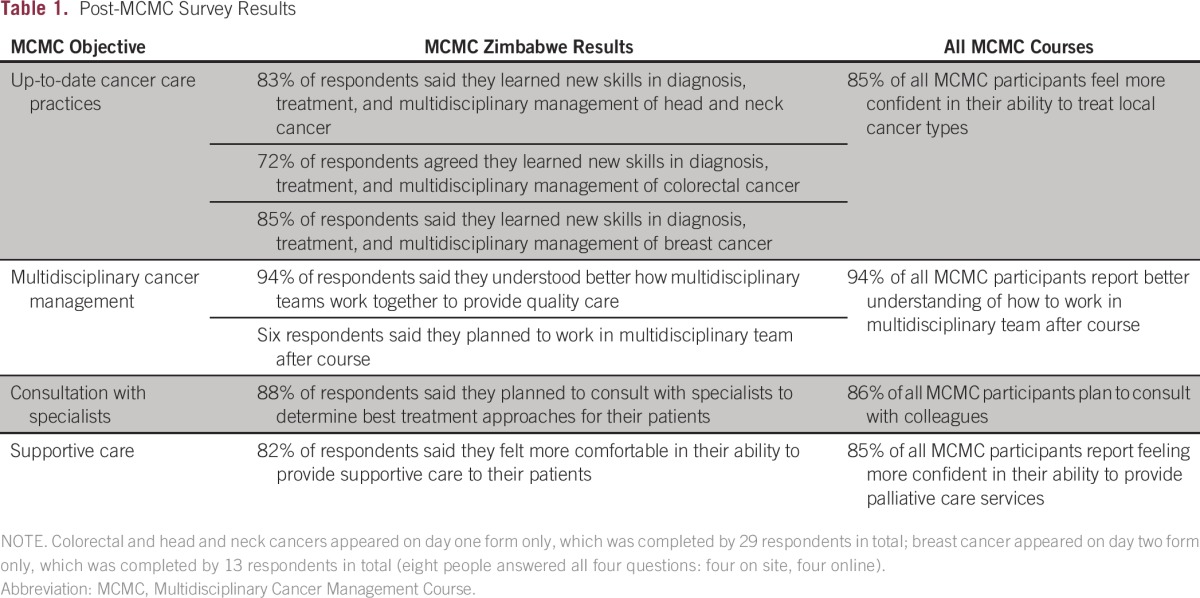
Post-MCMC Survey Results

Impact of the CourseLong-lasting partnerships and friendships were fostered at this course. The visitors appreciated the challenges faced by the local experts and also found a lot of common ground with the local experts. One expert is working with the Zimbabwean local cancer center to build a radiotherapy program that will provide patients with cancer rapid radiotherapy using intensity-modulated techniques. Currently, the cancer center has advanced radiation linear accelerators but lacks the critical software licenses to implement more sophisticated radiotherapy techniques. The local team is working with the expert to get the software. In so doing, it is hoped that state-of-the-art radiotherapy will be provided in a more cost effective manner and exposure of normal tissue to radiation damage will be minimized. A collaborative study is also under way with one of the experts on patients with breast cancer to assess hormone receptor status in the local community, because this is currently not being performed at the state hospital. Monthly international radiotherapy medical record rounds between a local institute and one of the expert centers are being established to exchange information and knowledge.

The course has resulted in a more motivated workforce. The existing multidisciplinary meetings, such as the weekly breast cancer clinic, are being attended regularly by multidisciplinary teams. A monthly pediatric multidisciplinary team meeting has also been established. Since the course was completed, there has been increased attendance by specialists at the tumor board meetings and increased consultation among specialties.

## THE FUTURE

The relationship between ASCO and the Association of Radiation Oncologists and Radiologists of Zimbabwe is ongoing. We hope to facilitate additional exchanges of information and knowledge between high-resource countries and Zimbabwe. These would include exchange programs, training programs, and twinning programs between institutions, where a local department would be paired with another oncology department abroad to exchange ideas and knowledge. ASCO programs, like the International Cancer Corps, where volunteer oncology professionals share their medical expertise with locals, would be of great benefit if implemented here in Zimbabwe.^45^

At this point, the main efforts are geared toward sustaining the benefits accrued through the course. The long-term influence of the course needs to be assessed, namely, whether there is potential to build on the fostered relationships to maintain and improve on the gained knowledge and experience. If such courses are found to yield sustained benefits, it may be reasonable to implement them on a regular, regional basis. However, critical evaluation is needed to identify what should be improved on in subsequent courses or, perhaps more importantly, what previously unidentified needs and opportunities were revealed by the course. For example, it might be beneficial to have more onsite joint clinic visits to instruct and aid in patient management issues, more time for interactive discussion on specific cancer topics, or regular online tumor-specific tumor boards or consultation sessions.

We plan to evaluate the course impact after 1 year. If a sustained impact is demonstrated, more such courses will need to be planned, possibly in several cities in the region, with a rotation schedule and with attendance from several different countries. Additional courses will be guided by the experience gained, with the aim of better serving local needs.

Our evaluation had some limitations. These include the small number of respondents to the postcourse assessments and the short duration of time in which measure demonstrable outcome.

In conclusion, the state of cancer care remains critical in LMICs, with barriers to reducing the burden of cancer mostly attributed to a lack of health care infrastructure, a lack of resources, and shortages in the workforce.^46^ Educational efforts together with collaborative partnerships may prove to be the most cost-effective strategy for a sustainable effect. Multidisciplinary care remains important in cancer care, but its impact in developing countries needs to be evaluated. Oncology professionals in Zimbabwe are hopeful that this experience of a multidisciplinary care course will help mitigate the challenges they face in the delivery of state-of-the-art cancer care.
